# The Unique Life Cycle of *Strongyloides stercoralis* and Implications for Public Health Action

**DOI:** 10.3390/tropicalmed3020053

**Published:** 2018-05-25

**Authors:** Wendy Page, Jenni A. Judd, Richard S. Bradbury

**Affiliations:** 1 Miwatj Health Aboriginal Corporation, Nhulunbuy, NT 0881, Australia; 2 Public Health and Tropical Medicine, James Cook University, Cairns, QLD 4870, Australia; 3 Centre for Indigenous Health Equity Research, School of Health, Medical and Applied Sciences, Central Queensland University, Bundaberg, QLD 4670, Australia; j.judd@cqu.edu.au; 4 School of Health, Medical and Applied Sciences, Central Queensland University, Rockhampton, QLD 4700, Australia; r.bradbury@cqu.edu.au

**Keywords:** *Strongyloides stercoralis*, strongyloidiasis, life cycle, public health, control, biology

## Abstract

*Strongyloides stercoralis* has one of the most complex life cycles of the human-infecting nematodes. A common misconception in medical and public health professions is that *S. stercoralis* in its biology is akin to other intestinal nematodes, such as the hookworms. Despite original evidence provided by medical and veterinary research about this unique helminth, many assumptions have entered the scientific literature. This helminth is set apart from others that commonly affect humans by (a) the internal autoinfective cycle with autoinfective larvae randomly migrating through tissue, parthenogenesis, and the potential for lifelong infection in the host, the profound pathology occurring in hyperinfection and systemic manifestations of strongyloidiasis, and (b) a limited external cycle with a single generation of free-living adults. This paper aims to review and discuss original research on the unique life cycle of *S. stercoralis* that distinguishes it from other helminths and highlight areas where increased understanding of the parasite’s biology might lead to improved public health prevention and control strategies.

## 1. Introduction

**Figure 1:**
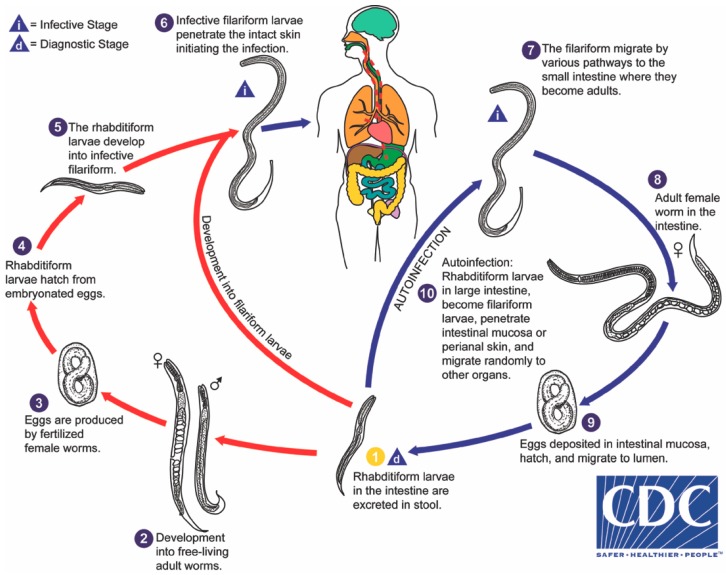
The life cycle of *Strongyloides stercoralis*. Distinctive features include (**a**) random migration of autoinfective larvae, (**b**) embryonated egg rapidly hatches to rhabditiform larvae, and (**c**) single generation of free-living male and female adults. Source: CDC DPDx: (https://www.cdc.gov/dpdx/), with permission.

*Strongyloides stercoralis* is distinguished amongst intestinal helminths by several factors of its biology, most impressively by its autoinfective life cycle (Figure 1), leading to potential lifelong infection and capacity to kill its human host, decades after initial infection. Strongyloidiasis affects an estimated 370 million people worldwide, based on data from 2013 [1]. Previously quoted estimates of 30 to 100 million people date back to 1989 [2]. The disease remains endemic in all tropical and sub-tropical countries worldwide, particularly in countries with developing infrastructure but also in developed nations such as the United States of America, Australia, Spain, and Italy [3]. Seroprevalence in some Latin American and African countries reaches in excess of 20% [4,5], and seroprevalence in excess of 40% has been reported in parts of South East Asia [6]. Rates of infection are concerningly high amongst refugees arriving from these countries when sensitive serological tests are employed, reaching 46% amongst Sudanese and 23% amongst Somalian refugees entering the USA in 2007 [7].

The reported prevalence in immigrants and refugees may, in fact, be an underestimate due to a reliance on insensitive microscopic methods for diagnosis. Single-dose albendazole is now a standard treatment for helminthic infection amongst refugees entering the United States. This treatment did reduce rates of strongyloidiasis in incoming refugees when tested by insensitive microscopic methods [8], although such methods do not detect very low–intensity infections. Albendazole treatment has a reported cure rate of 62–69%, depending on dosage regimen, as compared to 88–96% for ivermectin [9]. However, in many cases, these data were also based on insensitive faecal microscopic methods prior to the availability or routine application of faecal PCR and agar plate culture for the detection of larvae. Incomplete eradication of residual larvae after anthelmintic treatment resulting in recrudescence due to parthenogenesis and the autoinfective cycle needs more attention from researchers at this time.

Some developed countries retain limited, focal, endemic transmission of strongyloidiasis. Such environmental transmission often occurs in geographically limited foci or ‘hot spots’. Examples are remote Indigenous communities in Australia, where up to 59.6% of people tested seropositive for disease, with Indigenous and non-Indigenous residents being affected [10,11]. Studies of residents of rural East Tennessee in the late 1980s found a prevalence of 6.1% amongst hospitalized patients, almost certainly an underestimate given the insensitive microscopic method used for diagnosis [12].

Also, of particular concern to these developed countries is the prevalence of infection in travellers returned from highly endemic nations, with one study finding that *S. stercoralis* was the fifth most common clinical pathogen identified in returned international travellers presenting with infectious gastrointestinal disease [13]. Veterans of overseas conflicts are another important group in these countries at risk of long-term strongyloidiasis. A recent study of Australian Vietnam veterans found a *S. stercoralis* seropositivity rate of 11.6%, 40 years after the end of Australian involvement in that conflict [14]. The burden of disease in veterans of more recent conflicts and peacekeeping operations remains largely unexplored. These infections may present a hidden burden to the local health system many decades later if infected immigrants and returned travellers are immunosuppressed and develop severe systemic disease.

Developing strategies to reduce morbidity and mortality from strongyloidiasis in endemic areas requires a clear understanding of the idiosyncratic life cycle of *S. stercoralis*. Perhaps due to development of superficially similar rhabditiform into filariform stages of larvae at one point in its lifecycle, the overall biology of *S. stercoralis* is often confused with that of the hookworms, despite being very disparate. This paper highlights misunderstandings and errors in the interpretation of the parasite’s biology that may be hampering effective prevention, control, and eradication efforts worldwide.

## 2. Modes of Transmission to Human Host 

Transmission of infective larvae to the human host is almost exclusively depicted in scientific literature as being transdermal. Looss first demonstrated this process in 1904 by self-inoculating and infecting himself with *S. stercoralis* via exposing his skin to several hundred filariform larvae and subsequently finding larvae in his faeces 64 days after exposure [15]. Although not usually indicated in life cycle charts, and not the usual mode of transmission, experimentation by Wilms demonstrated oral ingestion of larvae by a human volunteer resulted in a shorter duration of 17 days before larvae were identified in faeces [16]. Transmission from human donor to human recipient can occur with organ transplantation [17].

## 3. The Internal Autoinfective Cycle—Ordered Pathway and Random Migration 

**Figure 2:**
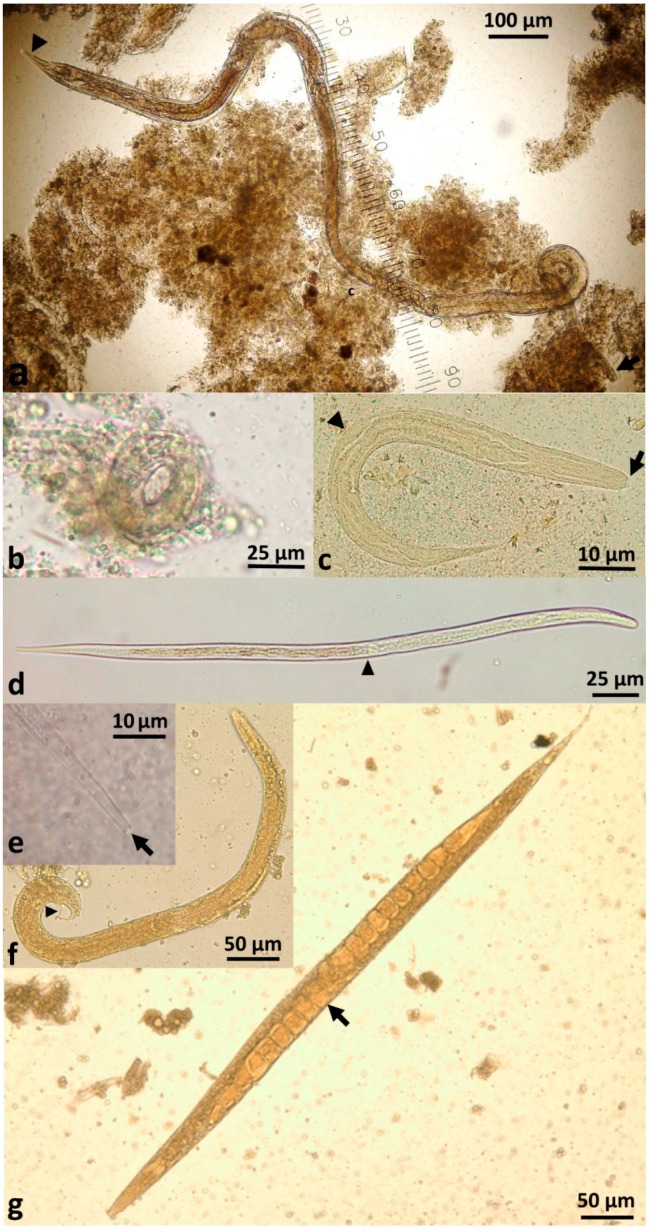
The life stages of *Strongyloides stercoralis*; (**a**) parasitic female with tapering anterior (arrow) and pointed caudal extremity (dart); (**b**) embryonated egg; (**c**) rhabditiform larva in faeces with short buccal cavity (arrow) and rhomboid genital primordium (dart); (**d**) filariform larva with oesophago-intestinal junction at mid-body (dart); (**e**) notched tail of the filariform larva (arrow); (**f**) free-living male with prominent spicule (dart); and (**g**) gravid free-living female with eggs in uterus (arrow). Note: Figure 2a,b are from faeces of a patient with hyperinfection; these life stages are not seen in patient faeces in the absence of severe hyperinfection. Figure attributions: (**a**,**b**,**e**): Dr Richard Bradbury, Central Queensland University; (**d**): Emeritus Professor John Goldsmid, University of Tasmania; (**c**,**f**,**g**): CDC DPDx web site (https://www.cdc.gov/dpdx/). Reproduced with permission.

*S. stercoralis* demonstrates remarkable persistence within the host due to its autoinfective cycle. The traditional pathway of infection is via infective filariform larvae entering the skin (transdermal) and being carried through the circulation to the right side of the heart and from there to the lungs. At this point, the larvae migrate through the alveoli, ascend the trachea and are coughed up and swallowed into the oesophagus. From there, larvae travel to the small intestine where they mature into parasitic female adults (Figure 2a). The parasitic females burrow into the lining of the gut and produce eggs that do not require fertilization from a parasitic male (parthenogenesis) and hatch in the mucosa. This blood-pulmonary pathway was described by Fülleborn in 1914, who examined the tracheae and oesophagi of transdermally-infected dogs [18]: ‘The first major studies were done in 1914 in tracheotomized dogs by Fülleborn (1914), who concluded that the majority of larvae passed via the bloodstream to the lungs, ascended the respiratory tree, were swallowed, then arrived in the small bowel where they completed their development’ [18,19].

Importantly, the blood-pulmonary pathway is not the only route by which *S. stercoralis* larvae may reach the human gut. Schad and his colleagues observed a lower number of larvae than would be expected in the lungs of dogs with massive hyperinfection, thus questioning the assumption that autoinfective larvae only followed the ordered traditional pulmonary-tracheal pathway [20]. Further studies monitored the pathways of migration of radiolabeled *S. stercoralis* larvae in 10-day-old pups [21]. This work demonstrated that larvae not only enter the gastrointestinal tract via traversing the trachea but also randomly migrate through the viscera and other tissue directly to the duodenum. In the findings of these studies, the authors considered random migration to be a significant part of the infective life cycle of *S. stercoralis* in dogs and likely to occur in other hosts, such as humans [21,22,23]. This work, whilst convincing and exquisitely performed, had not entered the medical literature [24], possibly due to the veterinary nature of the original study. The majority of current texts still only refer to a cardiopulmonary-tracheal migration phase for infective larvae.

Based on the migration of infective filariform larvae after cutaneous inoculation into dogs, it seems unlikely that the autoinfective larvae only follow an ordered route during chronic infection. The currently accepted assumption, that autoinfective larvae invading the tissues take the ordered pulmonary-tracheal pathway back to the small intestine and that random migration only occurs during disseminated infection, is unsupported by scientific studies. An exponential increase in numbers of *S. stercoralis* with dysregulation of the autoinfective cycle [24] resulting in multiple end-organ failure, are more likely to be the reason, rather than a change in migratory pathway.

Potential misunderstandings of larval migration have also led to the clinical manifestations of chronic strongyloidiasis being limited only to the skin, lungs and gastrointestinal tract, with extension to other organs then categorised under disseminated and hyperinfection. Biopsies and clinical specimens from the former three sites are generally easier to obtain. However, random migration throughout the organs may be occurring in limited numbers without disseminated disease or hyperinfection. This hypothesis explains the finding of *S. stercoralis* in ‘ectopic sites’ such as the parotid gland [25] and pericardial fluid [26] in cases of chronic strongyloidiasis, without apparent systemic disease. Recurrent meningitis without fatal dissemination [27,28,29] suggests the autoinfective larvae are transporting enteric bacteria on its random migratory pathway during chronic infection. Although categorised as a soil-transmitted helminth, the perception that *S. stercoralis* may benefit from broadening its category to a ‘tissue parasite’ [30] is supported by the apparent random migration of autoinfective larvae in the tissues, the burrowing of the parasitic female into the intestinal mucosa, and the common need for serologic evaluation for diagnosis. The more recent category of strongyloidiasis as a neglected tropical disease is warranted.

### Corticosteroids, Hyperinfection and Dissemination

Disseminated strongyloidiasis in humans has a very high case fatality rate, with 68.5% of 244 cases analysed in a recent systematic review having a fatal outcome [17]. If the increasing rate of prescriptions for corticosteroids and the relative lack of awareness of strongyloidiasis within the medical profession in first-world countries continues, *S. stercoralis* will likely become a growing clinical challenge, particularly in the context of increased immigration from and travel to highly endemic countries. Buonfrate and colleagues’ systematic review of 244 case reports (171 having at least hyperinfection and 73 with confirmed systemic disease) [17], found corticosteroid use was associated in 67% of all cases. Many of these patients had other sources of immunosuppression, such as leukaemia, organ transplantation, multiple myeloma, and cancer, but corticosteroids remain an outstanding common factor. Solid organ transplant recipients made up 11.5% of cases (10% having concomitant corticosteroid use) [17]. The fatality rate was 68% in this group. Ten percent of all studied cases carried human T-cell leukaemia virus type 1 (HTLV-1), a viral blood disease endemic in several areas of the world with high rates of strongyloidiasis, including Okinawa and Australian Indigenous communities [31,32], and associated with disseminated strongyloidiasis. Patients with HIV made up 13% of cases, with only 3% of those HIV-positive individuals also taking corticosteroids [17]. A handful of patients had alcoholism or severe malnutrition as underlying pathologies. An apparently healthy patient developed hyperinfection and died despite being given thiabendazole [17].

The close association of corticosteroids with hyperinfection, sometimes leading to systemic disease, may be due to more than just host immunosuppression. Observational studies noted a strong association between corticosteroids and disseminated strongyloidiasis and suggested that immunosuppression alone did not precipitate dissemination. Genta observed that, in many cases, patients developed massive disseminated disease within 10 days of receipt of corticosteroids, where no larvae were detectable in their faeces prior to beginning immunosuppressive therapy [24]. In one case report, a single sub-conjunctival injection of dexamethasone triggered severe disseminated disease [33]. Ectopic production of adrenocorticotropic hormone (ACTH), which stimulates secretion of glucocorticoids, and ACTH treatment in two patients, also led to disseminated disease [34,35]. Genta also noted that in many cases, patients do not present with other diseases aligned with immunosuppression, such as candidiasis, reactivation of cytomegalovirus or toxoplasmosis (Genta, 1992). He surmised that corticosteroid treatment promoted ecdysis (moulting) of rhabditiform larvae in the gut and transformation to autoinfective larvae, thereby rapidly increased helminth load via autoinfection and caused systemic disease [24].

More recent work has explored this hypothesis in the laboratory. The steroid/thyroid hormone receptor from filariform larvae of *S. stercoralis* [36] and the steroid hormone dafachronic acids (DAs), which regulate the growth of *Caenorhabditis elegans* nematodes, also determines dauer arrest or reproduction growth in *S. stercoralis* [37]. Treatment with glucocorticoids was found to be necessary to induce hyperinfection in mice even when they were already severely immunocompromised [38].

## 4. The Eggs of *Strongyloides stercoralis*—Rarely Seen

In contrast to other intestinal nematodes, *S. stercoralis* larvae, rather than eggs, are passed in faeces. The parasitic female produces thin-shelled, ellipsoid eggs at early cleavage stage, which rapidly embryonate (Figure 2b) and then hatch in the crypts of Lieberkühn in the intestinal mucosa [39]. Eggs are only passed in faeces in cases of severe hyperinfection.

Embryonated eggs of *S. stercoralis* have occasionally been found in bronchoalveolar aspirates of patients with systemic strongyloidiasis [1]. The presence of eggs from parasitic adults [40] in respiratory specimens is a poor prognostic sign, as eggs are rarely seen, and is indicative of severe, life-threatening infection.

## 5. Developmental Pathways after Hatching

The eggs of the parasitic female rapidly develop and emerge as first-stage rhabditiform larvae. These rhabditiform larvae (L1) migrate to the intestinal lumen, where they feed on their passage along the intestinal tract. There are three separate developmental pathways that the larvae may undergo.

1. Internal autoinfective cycle: first-stage rhabditiform larvae moult to second-stage rhabditiform and further moult to become autoinfective filariform (L3) larvae that do not leave the human host. These autoinfective larvae migrate through the host to become parasitic adult females living in the small intestine and producing further offspring by parthenogenesis. In this way, *S. stercoralis* remains in intimate contact with its host. This autoinfective cycle, a distinguishing feature of *S. stercoralis*, allows the worms to maintain infection for many decades in the human host, in one case lasting for up to 65 years [41]. As described above, this internal autoinfective cycle ensures long-term survival of this species independent of the external environment.

2. External direct or homogonic cycle: Larvae leave the human host via faeces to the external environment. First-stage rhabditiform larvae moult to second-stage rhabditiform larvae and moult a second time to become infective filariform third-stage larvae (L3i) (Supplementary Video S1). These active, non-feeding filariform larvae may survive in a suitable environment for up to two weeks [19,20] until finding a new host. This cycle is referred to as the direct external cycle or the homogonic cycle.

3. External indirect or heterogonic cycle: Larvae leave the human host via faeces. First-stage rhabditiform larvae undergo four moults to become (i) rhabditiform free-living adult females (Supplementary Video S2) or alternatively, (ii) rhabditiform free-living adult males [20].

These free-living adults are further part of the indirect external or heterogonic development cycle. They mate, and the females produce eggs that are passed and rapidly hatch to become rhabditiform larvae (Figure 2c). In senile older females, eggs will develop to larvae within the uterus and escape from the decaying mother’s body [42]. These rhabditiform larvae feed on bacteria from the faecally-contaminated soil and then moult as in the homogonic cycle to become infective, non-feeding filariform larvae (L3i) (Figure 2d). *S. stercoralis* larvae typically live for less than three weeks, even in soil under optimal conditions with a temperature of 20–28 °C and high moisture. Larvae die rapidly in unfavourable conditions, impeding faecal diagnostic tests in remote laboratories that rely on viable larvae [43]. This generation of filariform larvae are definitively unable to develop into free-living adults [44] (Figure 2f,g) and have only one goal—to find a human host, failing which they will die within two weeks or less in the environment [20]. The male and female free-living adult worms only live for 2–4 days [44,45,46]. This single free-living generation amplifies the number of infective filariform larvae in the environment seeking a human host [20,47]. The duration of time in the environment of the external cycle is limited to three weeks maximum in an optimum environment [20,30,46].

## 6. The Single Generation of the Free-Living Cycle

The false perception that *S. stercoralis* may survive in the environment in a cycle of unlimited generations of free-living adults may have been perpetuated by observations of morphologically-similar free-living rhabditoid nematodes from soil in culture. Speare noted in 1989 that ‘It is important to be able to differentiate rhabditoids from *Strongyloides*. Failure to do so has led to a number of authors presenting rhabditoids as evidence of recurrent free-living generations of *S. stercoralis*’ [48]. Faecal cultures can be easily contaminated by free-living rhabditoids from the perianal skin of patients even when collected directly into sterile containers [49]. Free-living environmental rhabditoid larvae may have then been confused with *S. stercoralis* upon culture.

Difficulties in differentiating between the *Strongyloides* species may also have contributed to this assumption. Yamada’s study comparing *S. planiceps* to *S. stercoralis* demonstrated the free-living cycle of *S. planiceps* had up to nine generations and confirmed that *S. stercoralis* had only one single generation [44].

## 7. Implications for Public Health Action

A significant barrier to implementing control programs for strongyloidiasis in endemic communities has been the persistence of an incorrect perception that *S. stercoralis* persists indefinitely in the environment. This misconception that the external free-living cycle is recurrent, and the perceived higher risk of re-infection, appears to have been a barrier to treating asymptomatic persons with chronic strongyloidiasis in endemic communities [50]. Conway, Lindo, Robinson, and Bundy (1995) provided hope for control of strongyloidiasis in endemic areas by restating that the heterogonic free-living cycle has only one single generation, is short-lived in the environment, and has a low transmission rate with a long-lived infection in the human host [46].

Despite the overwhelming evidence for only one external free-living generation, many of the newer life cycle images of *S. stercoralis* in reference texts and websites include a backward arrow to indicate that the external indirect cycle will continue indefinitely in the environment, potentially leading to misunderstandings about the capacity to control *S. stercoralis* in endemic areas. 

Access to clean water, footwear, and sanitation has been fundamental to preventing new cases of strongyloidiasis. Treating the human reservoir of infection has achieved success in reducing prevalence and preventing clinical complications in some community studies [30,51,52,53].

The parasitic adult only produces up to 40 eggs per day. Low and intermittent output of larvae is a major factor for the low sensitivity of faecal testing, especially during the chronic phase of infection. Thus, faecal testing alone cannot be relied on for diagnosis, estimates of prevalence, or determining cure [30,43].

*S. stercoralis* has been resourceful in survival strategies, evident from both the long-lived autoinfective cycle in human hosts and the amplification of larvae through a single generation of free-living adults. Thus, transmission through other hosts should be considered.

## 8. Animal Reservoirs of Infection?

The role of reservoir animals in the spread and dissemination of *S. stercoralis* has not been thoroughly considered. It has been established that dogs, cats, and some primates may carry natural infections, but the capacity of these to be transmitted to humans was until recently obscure. Two recent phylogenetic studies of *S. stercoralis* from humans and dogs have been published [54,55]. Both of these papers found two very distinct haplotypes of *S. stercoralis*, one exclusively infecting dogs and a second found to infect dogs and humans interchangeably [54]. The role of domestic and wild dogs, and possibly also domestic cats, in the transmission and maintenance of *S. stercoralis* infection within affected communities warrants further research.

## 9. Conclusions

Improved understanding of the unique life cycle of *S. stercoralis* will better inform prevention and control strategies to reduce the associated morbidity and mortality caused by this parasite. Breaking the life cycle of any parasite is the key to public health prevention, treatment, and control. Knowledge of the life cycle indicates that this can be done in two ways: first by preventing infection through effective sanitation, hygiene and possibly treatment of dogs, and second by eliminating the parasites in the human host.

The fact that the external life cycle is limited to a maximum of one generation means that there is not an ongoing source of infection in the soil. The infection can only be transmitted when the soil is contaminated by faeces from an infected person, or possibly, infected dogs. The internal autoinfective life cycle ensures indefinite ongoing infection, and this means that infected people are the reservoir of infection, not the soil. This implies that effective treatment of people not only rids them of the morbidity and potential mortality associated with the infection, but also breaks the life cycle.

The increasing prevalence rates of strongyloidiasis and its capacity to kill the human host decades after initial exposure indicates this neglected tropical disease warrants a priority response from health care providers including government and non-government agencies.

Developing strategies such as measuring prevalence, identifying infected populations, providing treatment before clinical complications arise, and reducing the human reservoir of infection in endemic communities are imperative. The possibility of the presence of strains of *S. stercoralis* shared between people and dogs in endemic communities requires further research. A community-led shared effort with a medical, environmental, and veterinary One Health approach to prevention and control of strongyloidiasis may be the best strategy yet to thwart this neglected tropical disease.

## Supplementary Materials

The following are available online at http://www.mdpi.com/2414-6366/3/2/53/s1. Video S1: Video of many *Strongyloides stercoralis* filariform (infective) larvae and one gravid free-living adult female on Koga agar plate culture, demonstrating the rapid, serpentine motility of the filariform larvae. Video S2: Close-up video of gravid free-living adult female *Strongyloides stercoralis* with highly motile filariform larvae and cleaved eggs in the background on Koga agar plate culture, demonstrating numerous eggs within the uterus and distinctive slow motility of this life stage.
